# Levelling Profiles and a GPS Network to Monitor the Active Folding and Faulting Deformation in the Campo de Dalias (Betic Cordillera, Southeastern Spain)

**DOI:** 10.3390/s100403504

**Published:** 2010-04-08

**Authors:** Carlos Marín-Lechado, Jesús Galindo-Zaldívar, Antonio José Gil, María Jesús Borque, María Clara de Lacy, Antonio Pedrera, Angel Carlos López-Garrido, Pedro Alfaro, Francisco García-Tortosa, Maria Isabel Ramos, Gracia Rodríguez-Caderot, José Rodríguez-Fernández, Ana Ruiz-Constán, Carlos Sanz de Galdeano-Equiza

**Affiliations:** 1 Instituto Geológico y Minero de España, C/ Alcázar del Genil, 4, 18006 Granada, Spain; E-Mail: a.pedrera@igme.es; 2 Departamento de Geodinámica, Universidad de Granada, 18071, Granada, Spain; E-Mails: jgalindo@ugr.es (J.G.-Z.); aconstan@ugr.es (A.R.-C.); 3 Departamento de Ingeniería Cartográfica, Geodesia y Fotogrametría, Universidad de Jaén, 23071, Jaén, Spain; E-Mails: ajgil@ujaen.es (A.J.G); mjborque@ujaen.es (M.J.B); mclacy@ujaen.es (M.C.L.); miramos@ujaen.es (M.I.R.); 4 Instituto Andaluz de Ciencias de la Tierra, CSIC, 18002, Granada, Spain; E-Mails: aclopez@ugr.es (A.C.L.-G.); jrodrig@ugr.es (J.R.-F.); csanz@ugr.es (C.S.G.-E.); 5 Departamento de Ciencias de la Tierra y del Medio Ambiente, Universidad de Alicante, 03080, Alicante, Spain; E-Mail: pedro.alfaro@ua.es; 6 Departamento de Geología, Universidad de Jaén, 23071, Jaén, Spain; E-Mail: gtortosa@ujaen.es; 7 Sección Departamental de Astronomía y Geodesia, Universidad Complutense de Madrid, 28040, Madrid, Spain; E-Mail: grc@mat.ucm.es

**Keywords:** seismogenic faults, large folds, active tectonics, precise levelling, GPS, western Mediterranean

## Abstract

The Campo de Dalias is an area with relevant seismicity associated to the active tectonic deformations of the southern boundary of the Betic Cordillera. A non-permanent GPS network was installed to monitor, for the first time, the fault- and fold-related activity. In addition, two high precision levelling profiles were measured twice over a one-year period across the Balanegra Fault, one of the most active faults recognized in the area. The absence of significant movement of the main fault surface suggests seismogenic behaviour. The possible recurrence interval may be between 100 and 300 y. The repetitive GPS and high precision levelling monitoring of the fault surface during a long time period may help us to determine future fault behaviour with regard to the existence (or not) of a creep component, the accumulation of elastic deformation before faulting, and implications of the fold-fault relationship.

## 1. Introduction

**Figure 1.**
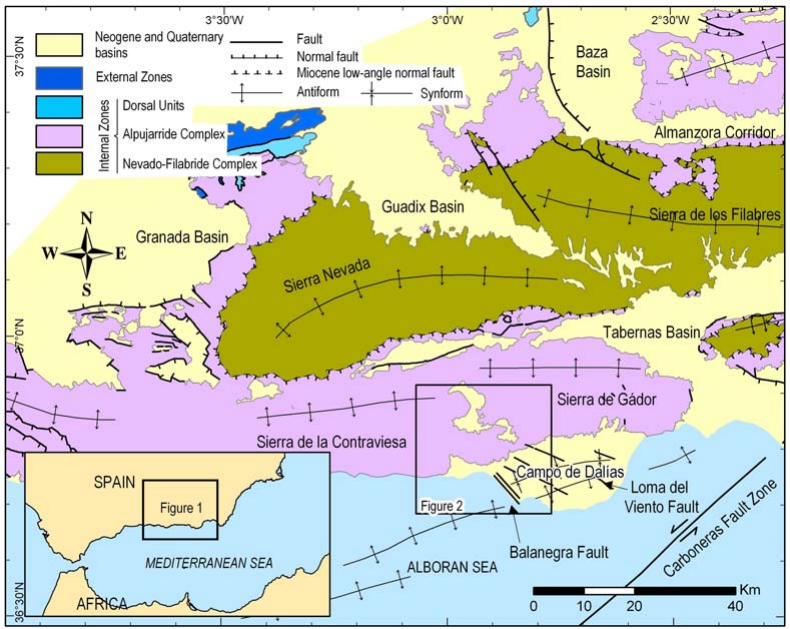
Geological map of the southeastern Betic Cordilleras.

The study of low rate deformation tectonic structures is of great relevance, because although they are related with high seismic risk, it is difficult to accurately determine the deformation rates. With most active tectonic studies focused on faults, the analysis of related fold development is poorly documented. The southeastern region of the Betic Cordillera (Figure 1), constituting one of the most seismically active areas within the Iberian Peninsula, is a prime target for such analysis. The current NNW-SSE convergence between the African and Eurasian plates mainly develops compressive structures such as large folds and strike-slip faults. Many NNW-SSE normal faults are coeval with compressive structures.

**Figure 2.**
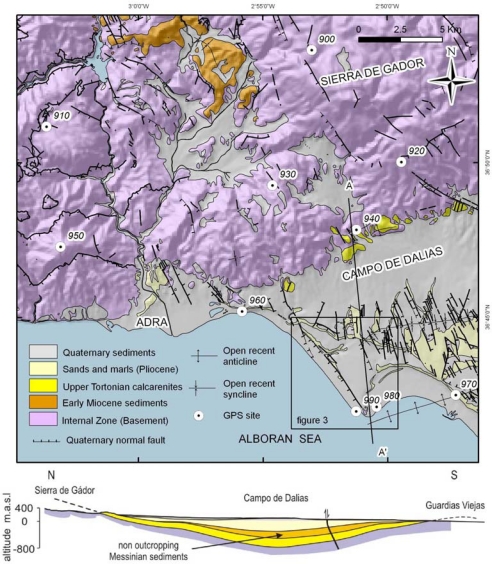
Geological map of the studied region. Location of GPS sites.

The Campo de Dalias and surrounding areas (Figures 1 and 2) comprise one of the most interesting zones for a characterization of present deformation as well as the relationship between the different compressive and extensional structures, folds and faults. The aim of this contribution is to describe the geologic, geomorphologic and seismic features related with recent tectonic activity, and to present data from the non-permanent GPS network and the high precision levelling profiles installed in this region. Horizontal NNW-SSE shortening through plate convergence and NE-SW coeval extension could thereby be determined, and the vertical uplift or subsidence related with development of folds and faults might be elucidated as well.

**Figure 3.**
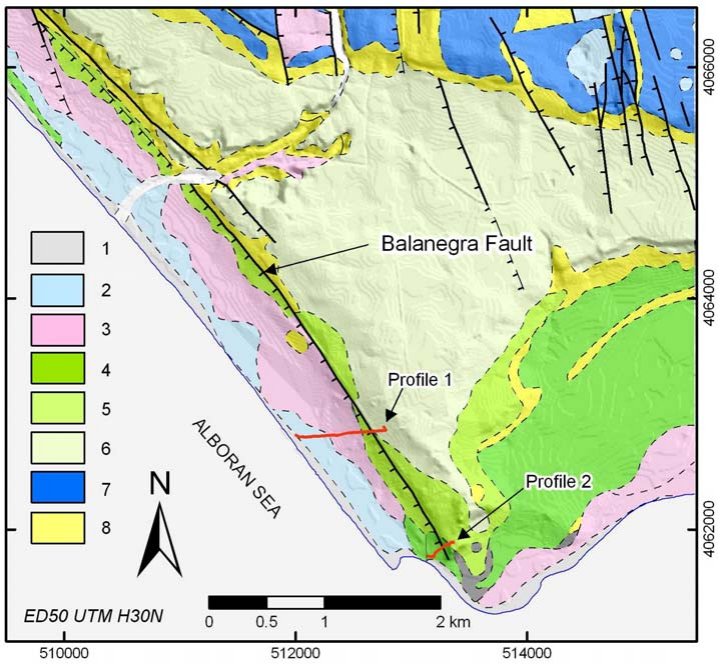
Detailed geological map of the southern end of Balanegra Faults including levelling profile location. 1: beach deposits (Holocene), 2: fluvial deposits (Holocene), 3: dunes (Holocene), 4: marine terrace (late Pleistocene), 5: marine terrace (middle Pleistocene), 6: marine terrace (early Pleistocene), 7: distal alluvial fans (Quaternary), 8: sands and silts (Pliocene).

In quantifying the deformation of a region, the establishment of GPS networks and levelling profiles prove adequate [1–3]. The best option is to use a permanent network of GPS stations; however, when this is not possible because of financial constraints, a network of closely-spaced pillars and its periodic re-observation through campaigns spaced over time is a very good alternative [4,5]. High precision levelling allows us to determine accurate vertical slips in the studied area. This study establishes a regional GPS network (Figure 2) and two levelling profiles across the Balanegra Fault, one of the faults showing most recent seismic activity in the zone (Figure 3). The benchmarks located in the hangingwall and footwall through the profiles permit assessment of its co-seismic activity with related elastic deformation or creep character, at least for the time period studied.

The combination of these two geodetic methods in repeated surveys will enhance our understanding of the relationship among fault movement and folding [2,3]. This research ultimately intends to answer the following questions: Is the deformation of folds and faults simultaneous? Is the fold development a result of aseismic movement? Can we accurately determine the uplift and slip rates? Can we use these data in the future for geological hazard assessment?

## 2. Recent Folds and Faults

The Betic Cordilleras (Figure 1) have undergone a polyphase evolution involving alternate and sometimes simultaneous compressional and extensional deformations since the early Miocene. However, the most recent tectonics, since late Miocene times, can be characterised by mainly NNW-SSE compression as a result of the Eurasian and African plate convergence. These plates converge at a rate of approximately 5 mm/y [6,7]. Coeval with compression, ENE-WSW orthogonal extension takes place. The NNW-SSE compression mainly develops large open folds with an ENE-WSW trend in a basin and range landscape in the surrounding mountains [8] (Figure 1). In the studied region, the reliefs of the Sierra de Gador correspond to a broad antiform that folds late Miocene calcarenites [9]. To the south, the Campo de Dalias plain is a large open synform having a WSW-ENE axis and an adjacent anticline southwards that gently folds late Miocene up to Quaternary sediments. This geometry in depth is well established from geologic, geophysical and well data (Figure 2), and reveals a growth fold//fold growth?? that continues southwards to the Alboran Sea. The offshore seismic profiles indicate that the most recent sediments are gently folded, so these folds might be active at present [9]. High-resolution sea floor imaging yields evidence of an active offshore rupture along a strand of the Carboneras Fault Zone in the Gulf of Almería, which suggests that these active and seismogenic faults may entail specific seismic hazards and tsunami potential [10].

The ENE-WSW coeval extension develops normal faults and tensional joints. The NW-SE normal faults also affect Quaternary sediments [11]. One of the most important faults within the area of study is the Balanegra Fault, featuring recent associated seismic activity. The Balanegra Fault zone comprises two main parallel faults along with other minor synthetic faults, producing a staircase morphology (Figure 3).

**Figure 4.**
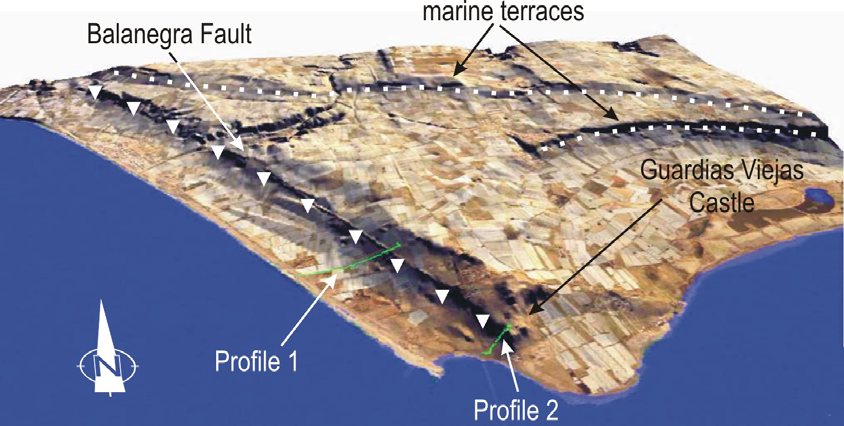
Digital terrain model of Balanegra Fault Zone showing the fault scarp and marine terraces (10 × 10 m). Vertical exaggeration has been applied for a better view.

The recent activity of these faults can also be inferred from geomorphological features, including evident topographic scarps in the landscape [12] (Figure 4). Furthermore, the coastline of the Campo de Dalías features some straight segments several kilometres long and a NW-SE trend that coincides with some of the principal faults, such as the Balanegra Fault, evidencing its recent activity. The difference in topographic height between the hangingwall and footwall of Quaternary normal faults reflects the minimum vertical throw. The vertical scarps have average heights of about 2 m, though they may reach 35 m (Loma del Viento Fault) or 38 m (Balanegra Fault), representing cumulative vertical throws.

## 3. Seismicity

The southern Betic Cordilleras are characterized by continuous shallow seismic activity of low to moderate magnitude, and less frequent large earthquakes that reveal the relatively high seismic hazard of this region. There are historical records of at least fifty destructive earthquakes, with detailed description of the main shocks, surface breaks and related damage distribution [13,14]. The village of Adra and its surroundings near the Balanegra Fault have undergone long periods of seismic activity, particularly in the past two centuries. The major earthquakes and their intensities, as documented near the Balanegra Fault, are shown in Table 1. For example, in 1804, an intensity of IX (MSK) was registered [15].

**Figure 5.**
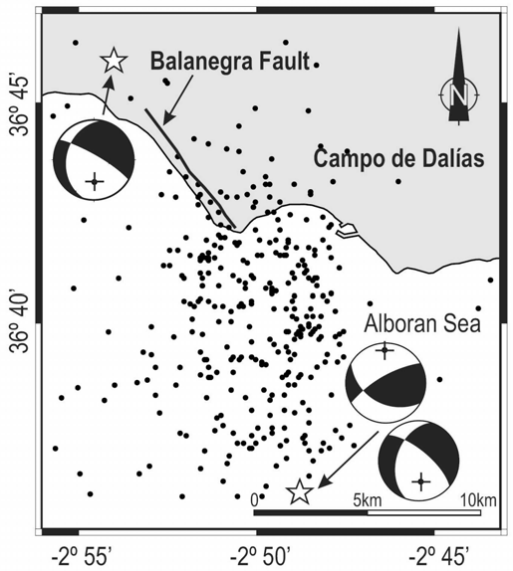
Epicentres and focal mechanisms near the Balanegra Fault.

The most intensive earthquake activity recorded recently in this region occurred from December 1994 to January 1995, including Mb (body wave magnitude) = 4.9 and Mb = 5.0 mainshocks (maximum intensity VII, MSK) [17]. Over the following three months, 350 events (Md (duration magnitude) ≥ 1.5) were recorded in the area, with most hypocentres at a depth between 0 and 12 km. The spatial distribution of seismicity defines an approximately NW-SE elongated region, limited to the North and South by the two mainshocks running parallel to the NW-SE Balanegra Fault (Figure 5). The focal mechanisms of the last two mainshocks show fault-plane solutions involving normal faulting with an oblique slip component [18] (Figure 5).

## 4. Uplift and Slip Rates

Several geologic data underline a clear uplift in the region since the late Miocene that suggests active tectonic deformation. In Tortonian times, a major transgression in the Betic Cordillera largely covered the region, and no previous relief can be seen [19]. Afterwards, the development of large folds and local normal faults caused either uplifting or the sinking of marine sediments, thereby generating the present relief [19,20]. The later sediments were deposited simultaneous to the fold and fault processes. Previous research efforts [19] calculated a minimum average uplift rate of 0.028 cm/yr for the highest peak in Sierra de Gádor, considering the absence of any relief in Tortonian times. Near the study area, at the northeastern boundary of the Sierra de Gádor, the drainage network shows incision rates between 0.03 and 0.07 cm/yr over the last 245,000 years, confirming the regional uplift of the area [21].

Meanwhile, Pleistocene marine terraces develop in the littoral, providing information about recent uplift of the region. Near the Loma del Viento Fault a total of 16 marine terraces have been described [22], forming a staircase profile rising up to 82 m above the actual sea level. The uplift rate in the upthrown block of the Loma del Viento over the last 130 ka is 0.0046 cm/yr [22], but uplift varies throughout the region because of interaction with the development of the folds and faults. Other marine terrace ages obtained near the Balanegra Fault [23] provide uplift rates of 0.009–0.012 cm/yr. Table 2 shows the uplift rates computed by several authors in previous works.

## 5. High Precision Levelling

The quantification of recent vertical movements through the comparison of high precision levelling data is a widely used technique for the monitoring of active faults and seismic deformations, since it determines vertical changes with great accuracy. The method is based on the comparison of changes in height measured in the levelling line in different surveys. Comparison of the levelling data provides information about the amount of deformation accumulated around the main fractures within the area of interest in the time span considered.

**Figure 6.**
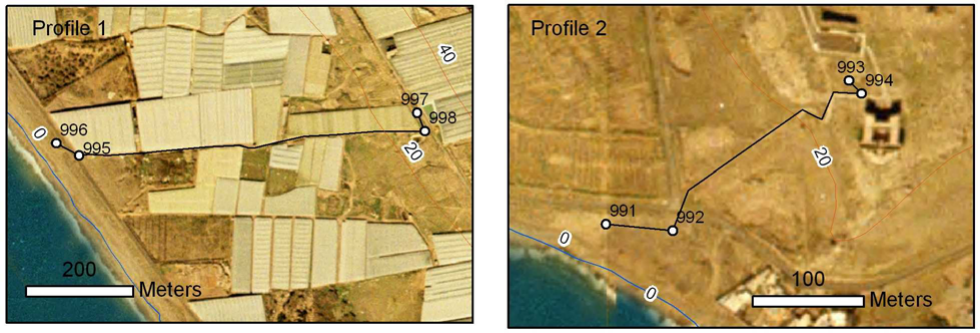
Detailed location of levelling profiles in the Balanegra Fault Zone.

This study shows the results obtained for two levelling profiles across the Balanegra Fault (Almeria, Spain) in two field campaigns, in 2006 and 2007 (Figure 3). The Greenhouses Profile (Profile 1, Figure 3) is 800 metres long and stretches from 997 to 996 benchmarks, whereas the Old Guards Fortress Profile (Profile 2, Figure 3) has a length of 350 metres and runs from 993 to 991 benchmarks (Figure 6). All benchmarks were embedded vertically on rocks to guarantee their stability.

The accuracy of levelling operations is dependent upon the quality of the instrument used and its adjustment [24]. A Leica DNA03 digital level, two 3-metre Invar bar-coded staffs, two sturdy steel-spiked base plates for a stable staff position on all types of terrain between benchmarks, and a tripod with fixed-length legs were used in the high-precision levelling campaigns. All data were saved automatically in the internal memory. In this way, the data could easily be uploaded into a computer. The manufacturer guarantees a standard deviation of ±0.3 mm/km of double levelling, but there are many factors affecting the accuracy of readings in the development of a high-precision levelling profile. Therefore it is necessary to consider, on the one hand, the staff calibration; and on the other hand, the method of operation with the level.

The measurements were taken automatically, free of the influence of the Earth’s curvature, due to an option activated on the digital level. The collimation error was calculated by means of the Förstner Method. The level automatically corrects all the measurements as well.

Each levelling run always started and finished with the same benchmark rod in order to avoid zero-point differences between rods. Every profile was observed back and forth. Allowable misclosures were computed by ±0.5 mm√k, where k is the total length levelled in kilometres. The levelling method used was BFFB (backsight—foresight—foresight—backsight). The time interval between readings was kept as short as possible. Likewise, backsight and foresight distances were kept as close to one another as possible, up to some centimetres per set-up, meaning that the total difference throughout the line was less than 10 cm. A staff scale range of 0.5 m to 2.5 m was used. In order to prevent atmospheric refraction errors, the line of sight was at no point closer to the ground than 50 cm. Temperature changes during the observation campaigns were minor. The standard deviation σ_Δz_ in mm of the height difference between every two benchmarks is obtained by:

(1) $$\sigma_{\Delta Z}=\pm \sigma_{\text{ISO}-\text{LEV}}\sqrt{k}$$

where σ_ISO–LEV_ is the experimental standard deviation of a 1-km double-run levelling (ISO 17123-2, Levels) and k the total length levelled in kilometres.

The standard deviation σ when different campaigns are compared is computed by:

(2) $$\sigma =\sqrt{{\left(\sigma_{\Delta Z_{\text{campaign}\;1}}\right)}^{2}+{\left(\sigma_{\Delta Z_{\text{campaign}\;2}}\right)}^{2}}$$

**Figure 7.**
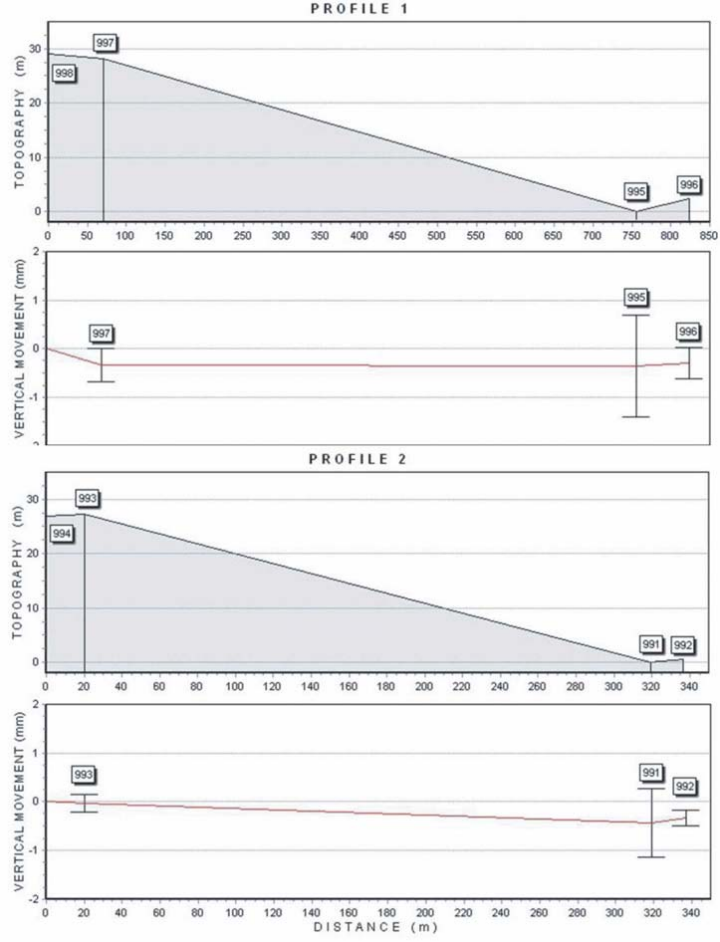
Recent vertical movement profiles in the Balanegra Fault Zone.

Two recent vertical movement profiles were constructed by comparing the benchmark height differences measured in the 2006 and 2007 campaigns by using the raw height differences between consecutive benchmarks obtained from the forward and the backward levelling data (Figure 7). In these profiles, the error bars are equal to two standard deviations with respect to the previous benchmark. As the error bars are larger than the vertical changes, the movements in this period are not significant in the two profiles.

## 6. GPS Network

For the purpose of quantifying deformation now occurring in the Sierra de Gádor and Balanegra Fault, the first non-permanent GPS network of this area was installed in 2006 (Figure 2). Ten sites form the geodetic network. Sites 900, 920, 930 and 940 are located in the Alpujarride Complex, in the Sierra de Gádor antiform area. Site 900 is atop the Sierra de Gádor mountain range, which corresponds to the antiform hinge, whereas sites 920 and 940 are on the southern limb. Sites 910 and 950 are located in high reliefs west of Sierra de Gádor. Between the two principal site groups, several NW-SE faults have been mapped and indicate recent activity, as Quaternary sediments are affected and fault scarps develop. Sites 960 and 990 are located near the coastline, in the hangingwall of Balanegra Fault, whereas site 980 is in the footwall. Site 970 is in the Campo de Dalías area near the antiform hinge.

The first survey was carried out in June 2006, tracking the GPS constellation throughout a five-day campaign, with 12-hour sessions over baselines with lengths from approximately 7 to 16 km. For data acquisition, nine dual frequency carrier phase GPS GX1230 receivers with LEIAX1202 antennas were used.

**Figure 8.**
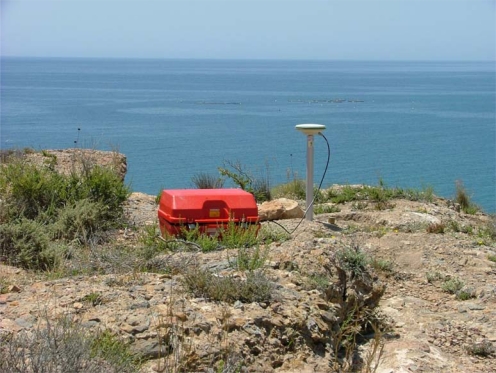
GPS site anchored to solid rock.

Each GPS site consists of a benchmark anchored to solid rock (limestones, conglomerates and metamorphic rocks). During measurements an aluminium tube of 0.5 m is screwed on with the GPS antenna located at the top (Figure 8). Only site 980 is a classical pillar. Simultaneous recording was done for five days.

The network was tied to the Rabat, San Fernando, Villafranca and Ebro sites, which belong to the IGS (International GNSS Service) network. From these IGS sites, the coordinates of the network central site, 930, were computed. The network solution finally computed for each day is a stochastically-constrained solution, based on the coordinates and their RMS of IGS sites. Then, ITRF2000 coordinates of the regional network at epoch 2006.4 were obtained by a least squares adjustment.

The GPS data processing was done using the Bernese 5.0 software [25]. The basic observables are dual-frequency GPS carrier phase observations. They were preprocessed in a baseline by baseline mode using triple differences. The preprocessing related to receiver clock calibration, performed by code pseudoranges, and detection and repair of cycle slips and removal of outliers, was carried out simultaneously for L1 and L2 data. During the final estimation, based on ionosphere-free double differences, a 10 degree elevation angle cutoff was used, and an elevation-dependent weighting scheme was applied. Precise ephemeris [26] and relative antenna phase centre variation files were used as recommended with ITRF2000. The a priori tropospheric refraction was modelled using the Dry-Niell [27] model; the remaining wet part was estimated hourly for all but one site, using the Wet-Niell mapping function [27] without a priori sigmas. Horizontal gradient parameters are estimated as well. Phase ambiguities which had been fixed to their integer values in a previous step are now introduced as known parameters. The strategy used to fix the ambiguities was QIF (Quasi Ionosphere-Free).

To these results we applied an intermediate program to produce GPS baselines with their covariance matrices. Afterwards the NETGPS software [28] was used to obtain a minimal-constrained network adjustment. NETGPS is a software package for analysing small and large networks established for high precision engineering surveys and geodynamic deformation control. This program performs the adjustment of GPS networks from the approximate coordinates of the network sites and a set of baselines with the covariance matrices estimated from processing the GPS phase and pseudorange observations. NETGPS estimates the station coordinates, both cartesian and ellipsoidal, and their covariance matrix along with the external reliability according to the Baarda theory by means of classical least squares adjustment. Table 3 gives the parameters of the minimum constrained adjustment.

Table 4 shows the adjusted geodetic coordinates in ITRF2000 at epoch 2006.4. It is important to stress that station 940 was not included in the processing because of problems with data recording.

## 7. Discussion and Conclusions

There is substantial geological evidence of recent and active deformation by folding and faulting in the eastern Betic Cordillera, which currently undergoes the NNW-SSE convergence of the Eurasian and African plates, while also affected by orthogonal extension. The dense fracture network is responsible for widespread seismic activity and is likewise responsible for the low to moderate magnitudes. If the fault length/magnitude relationships [29] and the maximum outcropping fault lengths are considered, earthquakes may reach up to 6 in magnitude. In any case, the presence of liquefaction structures provides evidence at least for the 5.5 magnitudes recorded [30]. In this framework, several faults like the Balanegra Fault have been shown to be the most active in view of related seismicity and geological features. Altogether, they permit characterization of the activity of these low-rate tectonic structures in widespread deformation zones, which have important related seismic hazards, but are nonetheless more difficult to constrain than the large high activity faults.

If we assume a relationship between the uplift rate and slip rate of the Balanegra Fault, it is possible to estimate the average recurrence interval. Firstly, a given displacement of 2.7 cm per event with magnitude 5 for the Balanegra Fault is assumed as a function of the fault length/magnitude [29] relationship. The uplift rates obtained [23] near the Balanegra Fault range from 0.009 to 0.012 cm/yr, so the recurrence interval obtained is 225 to 300 years. This is coherent with historical data, recording major earthquakes near the Balanegra Fault with VIII to IX intensities taking place in a time interval between 100 and 282 years. However, this a rough approximation, because the fault slips and time recurrence tend to be variable and do not follow a simple pattern.

The establishment of a non-permanent GPS network and two levelling profiles already measured affords an initial opportunity to monitor the present deformation and the relationships between fault and fold development. It is important to elucidate the fault behaviour, establish the maximum amount of elastic deformation before earthquake triggering, and determine if there is a creep slip component in addition to the seismic motion in order to more accurately assess the recurrence interval. However, due to the low activity rate, a long time span is needed to have accurate results with the present-day resolution of geodetic techniques. If we consider the uplift rates obtained by previous authors (Table 2) in conjunction with the accuracy of the GPS method applied, a minimum time span of 1.5 to 21 years would be necessary to measure significant vertical displacement. For the whole the Eurasian-African plate boundary, a regional convergence rate of 5 mm/yr [6] is detectable using the GPS method, with a one-year interval between two surveys. However, if this rate is distributed along the broad deformation zone, a longer time span is needed to determine deformation in the precise study area, affected by one of the most prominent and active faults of the Cordillera. The effect of horizontal WSW-ESE regional extension may be more easily determined, because the addition of several normal faults (accumulative heaves) could be more apparent and measurable than the effect of a single fault.

Two levelling profile surveys have thus far been measured, over a time span of one year. The results do not show significant movements, at least in view of the method′s accuracy. The [29] relationships between moment magnitude *versus* maximum displacement indicate a 0.027 m slip for an earthquake of magnitude 5, which was the last important earthquake involving the Balanegra Fault. Such coseismic slip, or creep, can be detected with this levelling method. The absence of significant vertical movement between the two surveys would suggest a purely seismic character for the Balanegra Fault, evidencing an interseismic period of accumulation of elastic energy and no slip on the main fault surface. Yet its long term activity is supported by geological features, and its seismic character is also constrained by the historical seismicity. In the future, levelling data after a medium magnitude earthquake would provide a good constraint for measuring the surface slip of this fault. Therefore, one of the conclusions to be underlined here is the seismic character of the Balanegra Fault.

In the study of these low-active tectonic structures, the advantages and disadvantages of geodetic methods, GPS and levelling, become evident. The levelling method is a powerful tool with high accuracy, able to detect small vertical slips. It would appear to constitute a good method for monitoring small or localized geological structures like the normal Balanegra Fault. The horizontal movements in this region of plate convergence are important, and they can be observed by GPS. Furthermore, in the case of large faults, both the elastic vertical and horizontal strain accumulated in the region between two main shocks can be detected using the GPS network.

The study of low-rate active tectonic structures, such as the Balanegra Fault and the Sierra de Gador Antiform, should be the main target of future hazard studies, because their seismogenic behaviour is capable of producing spaced high magnitude earthquakes. Their location right on the coastline makes them decisive as well for the constraint of possible tsunamigenic activity. At present however, a main concern regarding low-rate active folds and faults is that they require a very considerable time span to be studied, even with the highly accurate present-day geodetic techniques, a hindrance that must be overcome in the future for a better understanding of these regions with related seismic hazard.

## Figures and Tables

**Table 1.** Historical seismicity near Balanegra Fault (from [16]).

Date	Intensity	Location
22/09/1522	IX	South Adra
21/01/1804	VIII	Adra
25/08/1804	IX	Southwest Adra
16/06/1910	VII	Adra
16/06/1910	VIII	Adra

**Table 2.** Uplift rates from several authors in the studied region.

**Location**	**Uplift rate (cm/year)**	**Time interval**	**Reference**
Highest peak in Sierra de Gador	0.028 cm/yr	Late Tortonian—present (8 M.a)	[19]
Western part of Sierra de Gádor (rates of stream incision)	0.03–0.07 cm/yr	Pleistocene—present. Last 245,000 y	[21]
Marine terraces (Loma del Viento area)	0.0046 cm/yr	Late Pleistocene—present Last 130,000 y	[22]
Marine terraces (Guardias Viejas area)	0.009–0.012 cm/yr	Late Pleistocene—present Last 130,000 y	[23]

**Table 3.** Minimum constrained adjustment parameters.

Ses	Eq	Unk	Red	$\sigma_{0}^{2}$	${\overset{⌢}{\sigma }}_{0}^{2}$	$\chi_{\text{exp}}^{2}$	$\chi_{\mathrm{teo}}^{2}$	RMS X	RMS Y	RMS Z	*S_maj_*	*S_mim_*	CI
5	105	24	81	1	1.14	105.91	113.51	2	1	2	3	2	6

Ses: sessions, Eq: Number of equations, Unk: unknown parameters, Red: redundancy, $\sigma_{0}^{2}$ : a priori unit weight variance; ${\overset{⌢}{\sigma }}_{0}^{2}$: estimated unit weight variance; $\chi_{\text{exp}}^{2}$: experimental χ^2^ with “redundancy” degrees of freedom; $\chi_{\mathrm{teo}}^{2}$ theoretical χ^2^ with “redundancy” degrees of freedom at the 99% confidence level; RMS: root mean square in mm, *Smaj*: semimajor axis of the 99% confidence ellipse in mm. *Smin*: semiminor axis at the 99% confidence level in mm; CI: 99% mean value of the confidence height interval in mm.

**Table 4.** ITRF2000 geodetic coordinates at epoch 2006.4.

**Site**	**latitude [° ′ ″]**	**σ [m]**	**longitude [° ′ ″]**	**σ [m]**	**h [m]**	**σ [m]**
900	36 53 38.681421	0.001	−2 53 01.653521	0.001	1969.047	0.002
910	36 51 10.696921	0.001	−3 03 41.729952	0.001	919.580	0.003
920	36 50 01.555610	0.001	−2 49 25.894343	0.001	1109.671	0.003
930	36 49 16.842709	fixed	−2 54 36.596110	fixed	693.749	fixed
950	36 47 18.606936	0.001	−3 03 05.784845	0.001	935.216	0.002
960	36 45 12.459127	0.001	−2 55 50.264128	0.001	104.474	0.002
970	36 42 29.602625	0.001	−2 47 16.080250	0.001	133.799	0.002
980	36 42 07.044694	0.001	−2 50 26.764425	0.001	67.811	0.002
990	36 41 58.323988	0.001	−2 51 15.561034	0.001	53.565	0.002
